# Editorial: Current approaches to CRISPR/Cas9 delivery

**DOI:** 10.3389/fbioe.2022.1103007

**Published:** 2022-12-09

**Authors:** Hasan Uludağ, Hamidreza Montazeri Aliabadi, Giedrius Gasiunas

**Affiliations:** ^1^ University of Alberta, Edmonton, AB, Canada; ^2^ School of Pharmacy, Chapman University, Irvine, CA, United States; ^3^ Institute of Biotechnology, Life Sciences Center, Vilnius University, Vilnius, Lithuania

**Keywords:** CRISPR, viral delivery, non-viral delivery, gene editing, Cas9

Effective use of the CRISPR technologies requires a good understanding of the guide sequences, whose selectivity and efficacy have to be optimized to identify the right nucleotide sequences for editing. The enzymes capable of undertaking the genomic cleavage have to be similarly efficient and error-free when undertaking the required editing reactions. Very active and competitive research is underway to tackle these challenges and the biotechnology industry has spun several leading companies whose goal is to commercialize the full potential of CRISPR technology. Coupled with this endeavor, effective delivery systems are sought to transport the gene editing cargo to their site of action. The required cargo could be derived from different molecules, short oligonucleotides, long nucleic acid molecules derived from DNA or RNA, and active proteins ready to undertake enzymatic reactions. The diversity of the possible CRISPR enabling molecules will likely require different types of delivery systems that may have to be optimized for different cargo. Nevertheless, viral vectors have found initial utility to undertake the required delivery, given their broad applicability and efficient delivery mechanism as a result of evolutionary process, especially in the absence (or reduced) of safety concerns typical of cell culture and preclinical animal models. Some of the viral systems have been already translated into the clinical setting with careful attention to safety aspects of such a delivery mode. Non-viral approaches to delivery are taking flight as well to realize the full clinical potential of CRISPR, offering reduced safety concerns as compared to the use of viruses in patients.

This special issue has been compiled to highlight the recent developments in the CRISPR field. Six manuscripts have been compiled under the *“Current Approaches to CRISPR/Cas9 Delivery”* theme, originating from researchers in widely different geographic locations, including Russia, Ethiopia, China and United States. To provide a background on CRISPR and various opportunities, Shakirova et al. summarized the currently existing CRISPR/Cas9 applications with an eye on cell reprogramming. They have been compared with other non-CRISPR approaches and future perspectives and opportunities were highlighted. The review provides a glimpse into the evolution of cell programming technologies (as the basis of regenerative medicine) in the last decade, clearly laying out the components and mechanisms involved as the technology underwent evolutionary and engineered changes for improved performance. The authors furthermore provide a critical analysis of various approaches, providing an insight into the pertinent details of various approaches for CRISPR implementation.

As mentioned before, viral vectors have been the initial delivery choice to undertake CRISPR based editing and Mengstie provides a summary of various such vectors used in this approach. The possibility of using non-integrating adenoviruses (AVs) and adeno-associated viruses (AAVs) and integrating lentiviruses (LVs) have been succinctly laid out and various aspects of these viruses have been compared and contrasted. Given the availability of large numbers of effective viruses designed in recent years and the fast pace of recent advances in the field, other types of viruses and engineered variants of the more established viruses are bound to emerge in coming years. Rittiner et al. have further reviewed the rational design and construction of different types of designer molecules paired with viral-mediated cell delivery, specifically with LVs and AAVs. The focus of this review has been the utility of CRISPR/Cas system to bring about a desirable control over the pathological state of epigenetic regulation, and especially its use in the central nervous system (CNS). The authors nicely highlight the main concern with LVs, namely the possibility of insertional mutagenesis, with the aim of developing safer viruses to undertake the required delivery. The alternative, non-integrating AAV system was probed in sufficient detail to highlight its operational principles, concluding with a close look at several ongoing clinical studies with CRISPR/Cas technology. Both of these reviews provide a good glimpse into the main ‘workhorses’ that can enable implementation of CRISPR based editing.

Liang et al. further explored the delivery issue and summarized a broader spectrum of potential delivery systems, including physical methods (that are usually sufficiently effective under cell culture setting) and non-viral ‘chemical’ delivery systems that can be readily adopted for clinical use. With an eye on potential applications, the authors have laid out a broad range of delivery systems and highlighted the requirements as well as certain problems in CRISPR/Cas9 technology, such as the off-target effects, the efficiency of DNA repair mechanisms, and the delivery of the CRISPR/Cas system safely and efficiently to target locations. Better understand of these deficiencies are bound to create solutions that will improve the clinical translation of the technology. A complementary review of the CRISPR/Cas9 technology has been also provided by Foley et al. who summarize the current approaches to gene editing towards the development of emerging cellular therapeutics. Tools focusing on the two main components, namely the delivery vehicle and the gene editing cargo, have been probed from a bioengineering perspective. The identified barriers to biomedical applications were discussed related to efforts for widespread clinical translation. Physical methods are particularly emphasized in this review, whose clinical translation remains a challenge but can be exceptionally rewarding since no exogeneous agents need to be introduced into a host, provided that clinically acceptable means can be applied (such as ultrasound or mild electrical impulses) to a patient.

In an original contribution, Lv et al. reported on the feasibility of designing an inducible gene editing system that can be turned on on-demand. An all-in-one lentiviral system was described that can deliver both RNA-guided RNA endonucleases Cas13, which is equivalent to DNA-cleaving endonuclease Cas9 but employs RNA as the substrate, as well as the short CRISPR RNA (crRNA) to identify targets for editing. Reversible editing could be achieved as a function of doxycycline dose exposed to the cells, both in culture as well as in an animal model.

Collectively, the manuscripts in this research topic should help to propel the CRISPR/Cas technology towards better utilization of its potential. Thoughtful review papers as well as an original contribution from a diverse set of authors will be a valuable addition to the literature.

